# Analyzing Uncertainty in Complex Socio-Ecological Networks

**DOI:** 10.3390/e22010123

**Published:** 2020-01-19

**Authors:** Ana D. Maldonado, María Morales, Pedro A. Aguilera, Antonio Salmerón

**Affiliations:** 1 Data Analysis Research Group, University of Almería, 04120 Almería, Spain; 2 Department of Mathematics, University of Almería, 04120 Almería, Spain; maria.morales@ual.es (M.M.); antonio.salmeron@ual.es (A.S.); 3 Department of Biology and Geology, University of Almería, 04120 Almería, Spain; aguilera@ual.es; 4 Department of Mathematics and Center for the Development and Transfer of Mathematical Research to Industry (CDTIME), University of Almería, 04120 Almería, Spain

**Keywords:** Bayesian networks, entropy, socio-ecological system

## Abstract

Socio-ecological systems are recognized as complex adaptive systems whose multiple interactions might change as a response to external or internal changes. Due to its complexity, the behavior of the system is often uncertain. Bayesian networks provide a sound approach for handling complex domains endowed with uncertainty. The aim of this paper is to analyze the impact of the Bayesian network structure on the uncertainty of the model, expressed as the Shannon entropy. In particular, three strategies for model structure have been followed: naive Bayes (NB), tree augmented network (TAN) and network with unrestricted structure (GSS). Using these network structures, two experiments are carried out: (1) the impact of the Bayesian network structure on the entropy of the model is assessed and (2) the entropy of the posterior distribution of the class variable obtained from the different structures is compared. The results show that GSS constantly outperforms both NB and TAN when it comes to evaluating the uncertainty of the entire model. On the other hand, NB and TAN yielded lower entropy values of the posterior distribution of the class variable, which makes them preferable when the goal is to carry out predictions.

## 1. Introduction

Socio-ecological systems (SESs) constitute an outstanding example of complex systems, where multiple social and ecological components interact with each other in space and time [1,2]. SESs are complex adaptive systems whose interactions might change as a response to external events or endogenous changes [3,4]. As a consequence, the state of the SES evolves to a new one to adapt to these changes [5]. This brings about challenges not only from the modeling perspective but also when it comes to making predictions and diagnosing problems. An example of such complex socio-ecological systems is *cultural landscapes*, which are the outcome of the interaction of humans and nature over time [6]. Cultural landscapes [7] are typically heterogeneous systems providing diverse *ecosystem services* as the result of a complex relationship between human cultural management and the ecosystem.

Furthermore, there is a strong relationship between cultural landscapes and the socio-economy [8,9,10] and this relationship must be appropriately modeled in order to make well founded decisions on, for instance, implementing suitable landscape conservation policies [9]. Traditional analysis methods have been applied to this problem [11,12,13] but they sometimes fail to capture the complexity of the cultural landscape elements, connections and cause-effect relations, specially when ecosystem services are taken into account [14].

Another key issue is handling the uncertainty in data and in the predictions made by the models. In this sense, Bayesian networks (BNs) [15], provide a sound approach for handling complex domains endowed with uncertainty. The underlying formalism for uncertainty treatment is probability theory, which entails to quantify the uncertainty associated with the decisions made from BNs using measures as, for instance, Shannon entropy [16].

BNs have been widely used in the last decade as a modeling tool in environmental problems in general [17] and in cultural landscapes applications in particular [18]. A recent example employs the so-called object-oriented Bayesian networks (OOBNs) which are basically a structured way of representing Bayesian networks taking advantage of repeated and hierarchical components [19] so that the modeling task is simplified [20].

In this paper, we analyze the resulting model uncertainty when complex socio-ecological systems are modeled using Bayesian networks. More precisely, we investigate the impact of different network structures on the value of Shannon entropy from an experimental point of view. This analysis is relevant for practitioners when making decisions, since less uncertain models are potentially more reliable when making predictions using the model.

## 2. Materials and Methods

From now on, we will use uppercase letters to denote random variables and lowercase letters to denote a value of a random variable. Boldfaced characters will be used to denote random vectors (i.e., multidimensional random variables). The set of all possible values of a random vector X (also called its *support*) is denoted as $\Omega_{X}$. A *Bayesian network* [15] with variables $X=\{X_{1},\ldots ,X_{n}\}$ is a directed acyclic graph with *n* nodes where each one corresponds to a variable in X. Attached to each node $X_{i}\in X$, there is a conditional distribution of $X_{i}$ given its parents in the network, $Pa(X_{i})$, so that the joint distribution of random vector X factorizes as

(1) $$p\left(x_{1},\ldots ,x_{n}\right)=\prod_{i=1}^{n}p\left(x_{i}|pa\left(x_{i}\right)\right),$$

where $pa(x_{i})$ denotes a configuration of the values of the parents of $X_{i}$.

**Figure 1:**
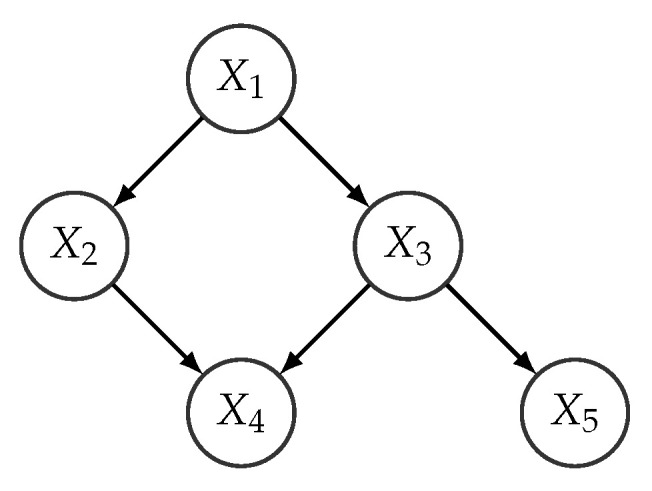
An example of a Bayesian network structure with 5 variables.

A simple example of a Bayesian network representing the joint distribution of variables $X_{1},\ldots ,X_{5}$ is shown in Figure 1. It encodes the factorization

(2) $$p\left(x_{1},x_{2},x_{3},x_{4},x_{5}\right)=p\left(x_{1}\right)p\left(x_{2}|x_{1}\right)p\left(x_{3}|x_{1}\right)p\left(x_{5}|x_{3}\right)p\left(x_{4}|x_{2},x_{3}\right).$$

From a modeling perspective, one advantage of Bayesian networks is that the induced factorization avoids the specification of large multivariate distributions that are replaced by a set of smaller ones, which are more easily specified, since the number of parameter is lower. For example, the factorization in Equation (2) replaces the specification of a joint distribution over 5 variables by the specification of 5 smaller distributions, each one of them with at most 3 variables. Another advantage is that the network structure describes the interaction between the variables in the model, in a way that can be easily interpretable.

One of the most successful areas of application of Bayesian networks is *classification* [21], which is a prediction task in which there is a discrete target variable *C*, called the *class*, whose value is to be forecasted from the values of a set of *feature* variables $X=\{X_{1},\ldots ,X_{n}\}$. The predicted value $c^{*}$ of *C* is computed as the one that maximizes the posterior distribution of *C* given the observed values of the features, that is,

(3) $$c^{*}=\arg\max_{c\in \Omega_{C}}p\left(c|x_{1},\ldots ,x_{n}\right).$$

**Figure 2:**
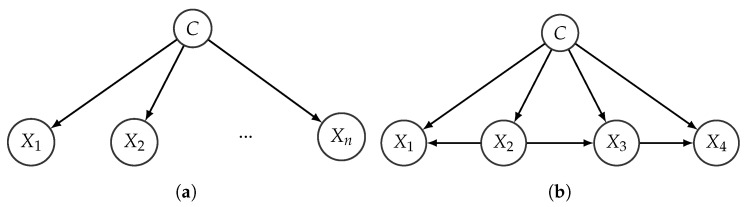
Structure of a naive Bayes model with *n* features (**a**) and a tree augmented network (TAN) model with 4 features (**b**).

Note that

(4) $$p\left(c|x_{1},\ldots ,x_{n}\right)=\frac{p\left(c\right)\times p\left(x_{1},\ldots ,x_{n}|c\right)}{p(x_{1},\ldots ,x_{n})}\propto p\left(c\right)\times p\left(x_{1},\ldots ,x_{n}|c\right),$$

which means that solving the classification problem requires the specification of an *n*-dimensional distribution for $X_{1},\ldots ,X_{n}$ given *C*. The problem can be simplified by representing the joint distribution using a Bayesian network and taking advantage of the factorization encoded by its structure. The strongest simplification is achieved when the network is forced to adopt a naive Bayes (NB) structure, where the feature variables are assumed to be conditionally independent given the class. The BN structure is depicted in Figure 2a.

Adopting an NB structure actually means a strong independence assumption, but in practice it is compensated by the low number of parameters that need to be specified. Notice that, in this case the factorization results in

(5) $$p\left(c|x_{1},\ldots ,x_{n}\right)\propto p\left(c\right)\prod_{i=1}^{n}p\left(x_{i}|c\right),$$

meaning that *n* one-dimensional conditional distributions must be specified, instead of one *n*-dimensional conditional distribution.

The independence assumption underlying NB models can be relaxed, resulting in more expressive models that still keep a reduced number of parameters. This is the motivation of the *tree augmented network* (TAN) structure [21], where each feature variable is allowed to have another feature as a parent, besides the class, as long as the resulting subgraph containing the features is a tree (i.e., it contains no directed cycles). An example of a TAN model is given in Figure 2b, corresponding to the factorization

(6) $$p\left(c|x_{1},\ldots ,x_{n}\right)\propto p\left(c\right)p\left(x_{1}|x_{2},c\right)p\left(x_{1}|c\right)p\left(x_{3}|x_{2},c\right)p\left(x_{4}|x_{3},c\right).$$

Given that there are multiple structures that one can choose when facing classification problems, ranging from NB to unrestricted Bayesian networks, a natural question is to know whether this choice has an impact on the performance of the classification model. This problem has been analyzed from the point of view of the accuracy of the classification model [21]. In this paper we are more interested in analyzing the impact of the model structure on the uncertainty over the predictions, which in this context can be evaluated as the uncertainty of the used Bayesian network.

After all, a Bayesian network represents a probability distribution and a well known approach to quantifying the uncertainty of a probability distribution is to use Shannon entropy [16]. The Shannon entropy of a discrete random variable *X* is

(7) $$H\left(X\right)=-\sum_{x\in \Omega_{X}}p\left(x\right)\log p\left(x\right).$$

Analogously, it can be defined over a random vector $X=\{X_{1},\ldots ,X_{n}\}$ as

(8) $$H\left(X\right)=-\sum_{x\in \Omega_{X}}p\left(x\right)\log p\left(x\right),$$

which in the case of a Bayesian network can be written as

(9) $$\begin{array}{ccc} H_{\mathrm{BN}}\left(X\right) & = & -\sum_{x\in \Omega_{X}}\prod_{i=1}^{n}p\left(x_{i}|pa\left(x_{i}\right)\right)\log\prod_{j=1}^{n}p\left(x_{j}|pa\left(x_{j}\right)\right)\\ & = & -\sum_{x\in \Omega_{X}}\prod_{i=1}^{n}p\left(x_{i}|pa\left(x_{i}\right)\right)\left(\sum_{j=1}^{n}\log p\left(x_{j}|pa\left(x_{j}\right)\right)\right). \end{array}$$

Particularly, for a Bayesian network with NB structure and variables $X=\{C,X_{1},\ldots ,X_{n}\}$, the entropy can be computed as

(10) $$H_{\mathrm{NB}}\left(X\right)=-\sum_{x\in \Omega_{X}}p\left(c\right)\prod_{i=1}^{n}p\left(x_{i}|c\right)\left(\log p\left(c\right)+\sum_{j=1}^{n}\log p\left(x_{j}|c\right)\right).$$

Shannon entropy is usually preferred to other entropies as a measure of uncertainty within the context of Bayesian networks due to its decomposability properties, which allow to efficiently compute it by taking advantage of the factorization of the distribution induced by the Bayesian network.

### 2.1. Experimental Analysis

In order to study the impact of the Bayesian network structure on the model uncertainty, we have conducted an experiment taking as a basis a Bayesian network that models a complex socio-ecological system. More precisely, we use the network described in [20]. It models the entire region of Andalusia (southern Spain) which contains a wide variety of scenarios from an ecological point of view.

The variables in the network describe social and economic indicators taken from the Multiterritorial Information System of Andalusia (SIMA) (http://www.juntadeandalucia.es/institutodeestadisticaycartografia/sima/) as well as environmental information collected from the Andalusian Environmental Information Network (http://www.juntadeandalucia.es/medioambiente/site/rediam). The network contains a total of 75 variables, described in the on-line material (https://w3.ual.es/personal/amg457/Downloads_protected/Experimentos.zip).

We conducted two experiments:

#### 2.1.1. Experiment 1

**Figure 3:**
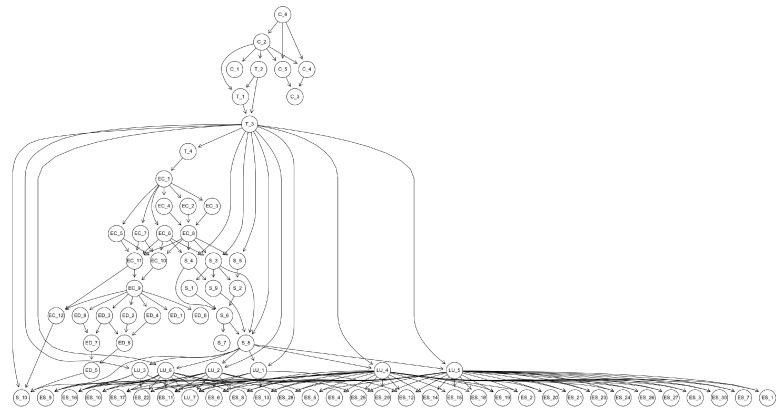
Structure of the Bayesian network used as reference in the experiments.

The goal of this experiment is to assess the impact of the Bayesian network structure on the entropy of the model. The starting point was the Bayesian network in [20], that will be referred to as Original BN. Its structure is displayed in Figure 3 and it gives an idea of the complexity of the described system. Out of Original BN, we generated samples of sizes ranging from 500 to 100,000. From each sample, we constructed 9 networks with NB structure, each one of them with a different class variable, 9 networks with TAN structure, with the same class variables as NB and 1 network where we imposed no restriction on its structure. NB and TAN networks were built using package bnlearn in R [22] while the other network was constructed using the greedy search (GSS) method implemented in Hugin (http://www.hugin.com).

Instead of computing the entropy of each of the obtained networks using Equations (9) and (10), we decided to estimate them. The reason is that a straight application of those formulas requires summing over a number of terms that grows exponentially with the number of variables. For instance, in the case of Original BN, that contains 75 variables, assuming that all of them had only 2 possible values, evaluating the entropy would require summing over $2^{75}$ terms (approximately $3.8\;\times \;10^{22})$.

The estimation of the entropy was carried out using the same samples utilized for constructing the Bayesian networks. For a sample of size *m*, $\left\{x^{\left(1\right)},\ldots ,x^{\left(m\right)}\right\}$, we estimated $H_{\mathrm{BN}}\left(X\right)$ as

(11) $${\hat{H}}_{\mathrm{BN}}\left(X\right)=-\frac{1}{m}\left(\sum_{j=1}^{n}\log p\left(x_{j}^{\left(r\right)}|pa\left(x_{j}^{\left(r\right)}\right)\right)\right),$$

where $x_{j}^{\left(r\right)}$ denotes the value of variable $X_{j}$ in the *r*-th element of the sample and $pa(x_{j}^{\left(r\right)})$ is the value of the parent variables of $X_{j}$ in the *r*-th element of the sample.

Similarly, we estimated $H_{\mathrm{NB}}\left(X\right)$ as

(12) $${\hat{H}}_{\mathrm{NB}}\left(X\right)=-\frac{1}{m}\left(\log p\left(c^{\left(r\right)}\right)+\sum_{j=1}^{n}\log p\left(x_{j}^{\left(r\right)}|c^{\left(r\right)}\right)\right).$$

Note that ${\hat{H}}_{\mathrm{BN}}\left(X\right)$ and ${\hat{H}}_{\mathrm{NB}}\left(X\right)$ are, respectively, unbiased estimators of $H_{\mathrm{BN}}\left(X\right)$ and $H_{\mathrm{NB}}\left(X\right)$. It can be easily proved taking into account that

$$\begin{array}{ccc} H_{\mathrm{BN}}\left(X\right) & = & -\sum_{x\in \Omega_{X}}\prod_{i=1}^{n}p\left(x_{i}|pa\left(x_{i}\right)\right)\left(\sum_{j=1}^{n}\log p\left(x_{j}|pa\left(x_{j}\right)\right)\right)\\ & = & E_{p}\left[-\sum_{j=1}^{n}\log p\left(X_{j}|pa\left(X_{j}\right)\right)\right], \end{array}$$

where $E_{p}$ denotes the expectation computed with respect to distribution $\prod_{i=1}^{n}p\left(x_{i}|pa\left(x_{i}\right)\right)$. Therefore, ${\hat{H}}_{\mathrm{BN}}\left(X\right)$ is just the sample mean estimator of $H_{\mathrm{BN}}\left(X\right)$, which is known to be unbiased. Likewise, ${\hat{H}}_{\mathrm{NB}}\left(X\right)$ is the sample mean estimator of $H_{\mathrm{NB}}\left(X\right)$. Since both estimators are unbiased, their accuracy can be measured using their variance or equivalently, their standard deviation, as variance coincides with mean squared error for unbiased estimators.

#### 2.1.2. Experiment 2

In this experiment we used the same networks as in Experiment 1. Then we generated three scenarios in the socio-ecosystem described by the Bayesian network. Each scenario corresponds to a particular configuration of values of some variables in the network. For each scenario, we computed the *posterior* distribution of the class variable—see Equation (4)—from each one of the nine networks in Experiment 1 and estimated the entropy of the posterior distribution as we describe next. The *prior* distribution of the class variable corresponds to the marginal distribution of variable *C* in the corresponding network in Experiment 1, without taking into account the data corresponding to the three scenarios analyzed here. This is equivalent to adopting a parametric empirical Bayes approach, where the parameters of the prior distribution are estimated by maximum likelihood. This is the usual way of approaching prediction problems with Bayesian networks when we have an initial sample with a high number of elements and without missing values. If we denote by q(c) the posterior distribution of the class variable for one particular scenario, then the entropy in this experiment is calculated as

(13) $$H\left(C\right)=-\sum_{c\in \Omega_{C}}q\left(c\right)\log q\left(c\right).$$

Note that in this case there is no need to estimate the entropy from the sample, as we only need to sum over the values of the class variable.

## 3. Results and Discussion

**Figure 4:**
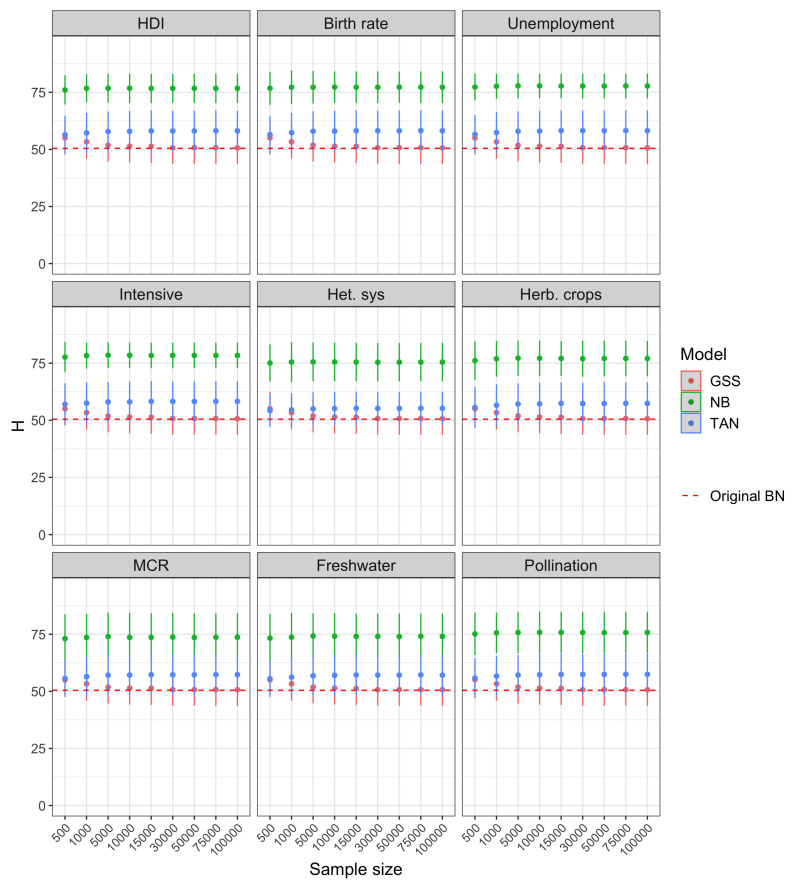
Shannon entropy vs. sample size for the Bayesian networks used in Experiment 1.

The results of Experiment 1 are reported in Figure 4. The dashed line corresponds to the Original BN, that constitutes the ground truth. The dots represent the estimated entropy values, while the bars centered on each point represent the standard deviation, and thus the accuracy of the estimated value. It can be seen how in this case the network with unrestricted structure (GSS), consistently outperforms both NB and TAN. In fact, the entropy of the GSS network converges to the exact one (Original BN) when the sample size increases. Focusing on the classification-oriented networks, the uncertainty is clearly lower (lower entropy) for TAN compared to NB. This comes to no surprise, as the structure of the NB is the most simple one and therefore it is more unlikely that it is able to capture the exact model accurately and this is reflected in the model uncertainty. In the case of NB and TAN, the increase in sample size does not lead to a reduction in the entropy. This is also consistent with the lack of ability to fit the right model of both structures, due to the independence assumptions.

**Figure 5:**
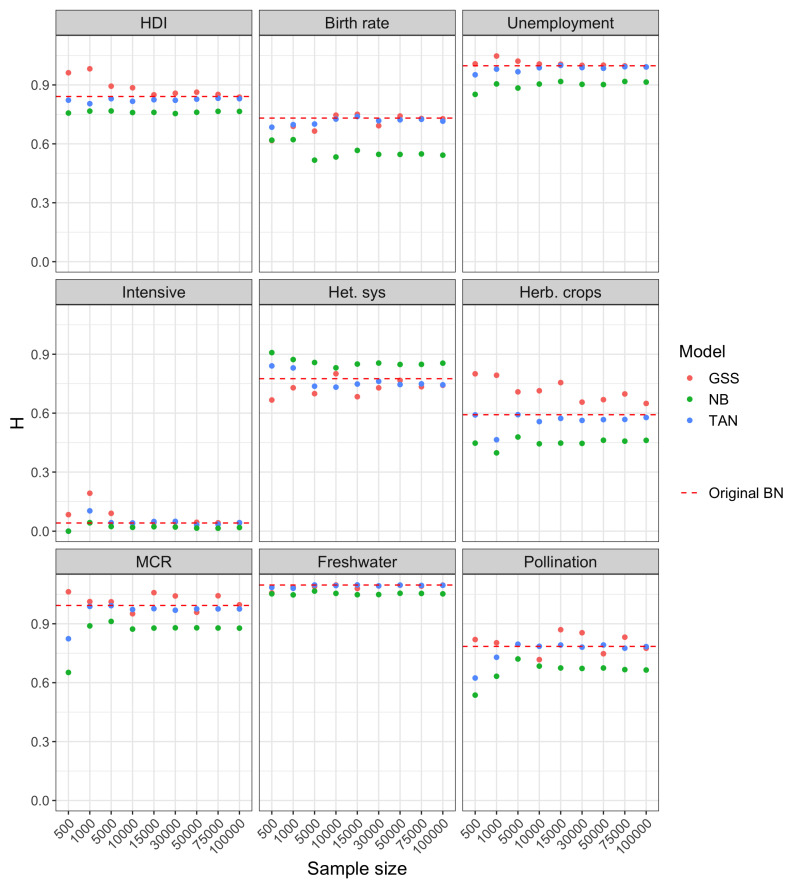
Shannon entropy of the class posterior distribution vs. sample size for scenario 1 in Experiment 2.

**Figure 6:**
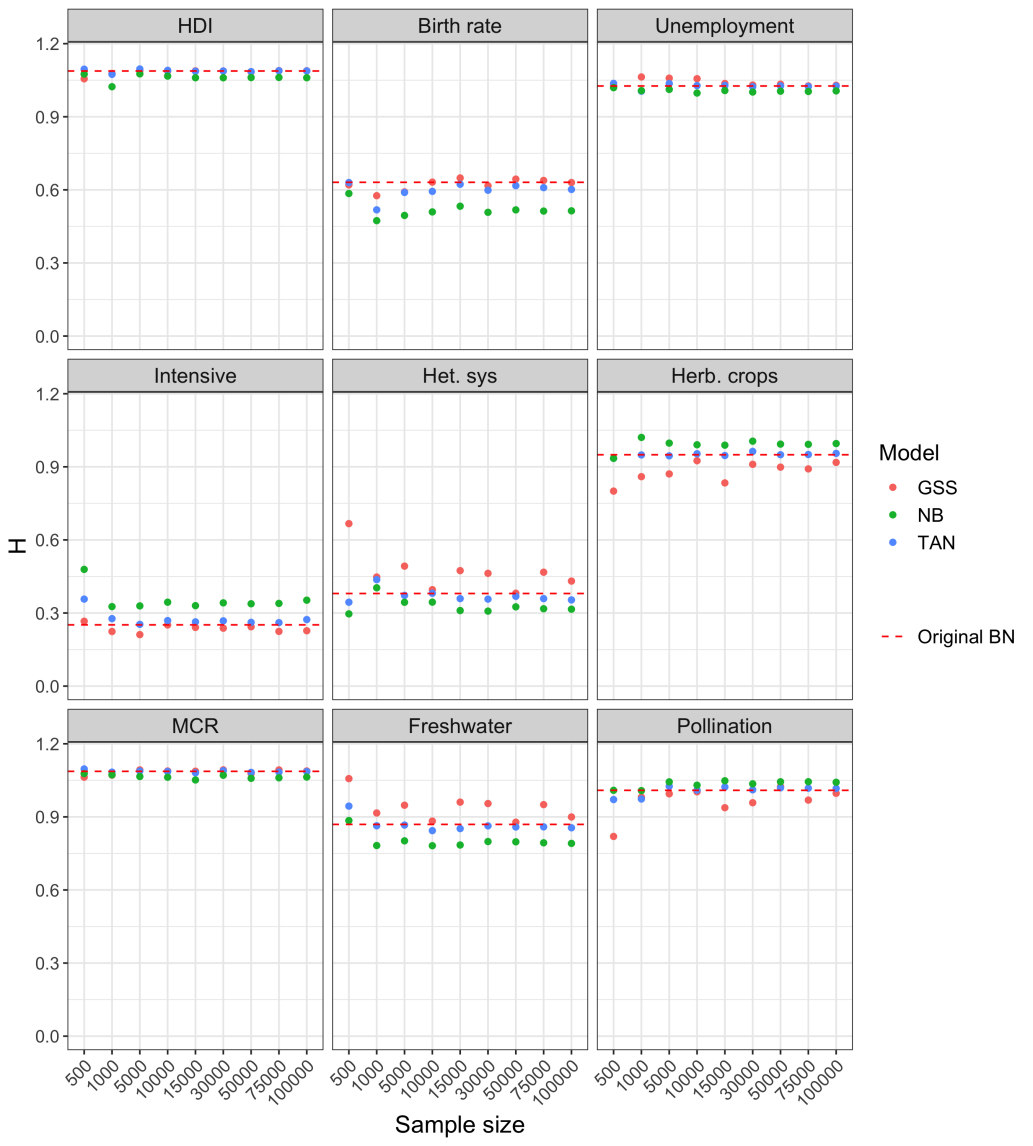
Shannon entropy of the class posterior distribution vs. sample size for scenario 2 in Experiment 2.

**Figure 7:**
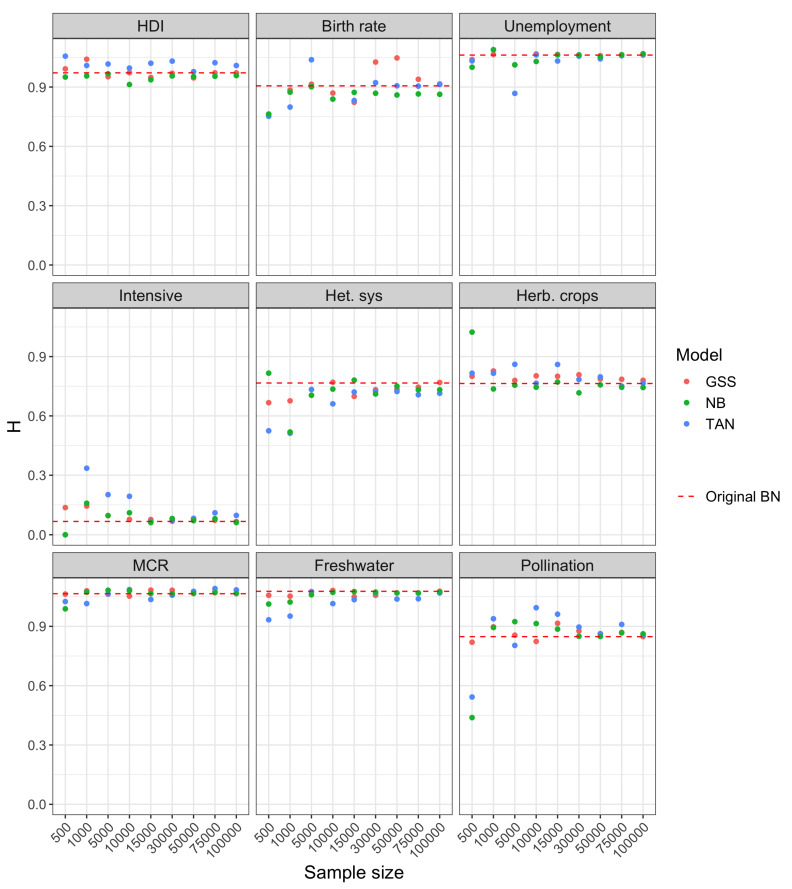
Shannon entropy of the class posterior distribution vs. sample size for scenario 3 in Experiment 2.

With respect to Experiment 2, the results for the three scenarios considered is similar, as can be inferred from Figure 5, Figure 6 and Figure 7. The comparison carried out in this experiment is more fair with respect to NB and TAN because it refers to prediction scenarios, in which case we are only interested in the distribution over the target variable and not the entire model. In the three scenarios, the entropy corresponding to NB and TAN, likewise GSS, also converges to the entropy of the class posterior distribution computed with the original network.

For smaller sample sizes, the uncertainty of GSS is typically higher than the exact one, which is in-line with the result obtained in Experiment 1 for this network. However, the uncertainty of the class posterior obtained from NB and TAN structures is often below the entropy of the Original BN and, in general, clearly below the uncertainty obtained from GSS. The extreme case is the posterior of variable MCR in scenario 1 computed from NB (bottom left panel of Figure 5).

The observed behavior of the analyzed models support the idea of using NB and TAN for classification instead of unrestricted Bayesian network structures. The fact that the uncertainty is lower means that the class posterior distribution is less smooth. In other words, it better discriminates the most probable value of the class, which is in fact the value that corresponds to the outcome of the prediction model, as seen in Equation (3). This is precisely the effect that is sought by NB and TAN, which are focused on being accurate in the predictions rather than in goodness of fit.

## 4. Conclusions

In this paper we have carried out two experiments analyzing the uncertainty in various Bayesian network structures representing complex environmental networks. More precisely, we have tested unrestricted structure, NB and TAN models representing a complex socio-economic system with 75 variables.

According to the results of the experiments, the conclusion is that, from the point of view of uncertainty, unrestricted structures are preferable when the goal is the representation of the entire complex system, that is, the full model. However, if the goal is to carry out predictions, then NB and TAN yield less uncertain results.

## References

[1] Liu J., Dietz T., Carpenter S.R., Folke C., Alberti M., Redman C.L., Schneider S.H., Ostrom E., Pell A.N., Lubchenco J. (2007). Coupled human and natural systems. AMBIO.

[2] Rescia A., Pérez-Corona M.E., Arribas-Ureña P., Dover J.W. (2012). Cultural landscapes as complex adaptive systems: The cases of northern Spain and northern Argentina. Resilience and the Cultural Landscape: Understanding and Managing Change in Human-Shaped Environments.

[3] Parrott L., Meyer W.S. (2012). Future landscapes: Managing within complexity. Front. Ecol. Environ..

[4] Ropero R.F., Rumí R., Aguilera P.A. (2016). Modelling uncertainty in social-natural interactions. Environ. Model. Softw..

[5] Schlüter M., Haider L.J., Lade S.J., Lindkvist E., Martin R., Orach K., Wijermans N., Folke C. (2019). Capturing emergent phenomena in social-ecological systems. Ecol. Soc..

[6] Blondel J. (2006). The design of Mediterranean landscapes: A millennial story of humans and ecological systems during the historical period. Hum. Ecol..

[7] García-Llorente M., Martín-López B., Iniesta-Arandia I., López-Santiago C.A., Aguilera P.A., Montes C. (2012). The role of multi-functionality in social preferences toward semi-arid rural landscapes: An ecosystem service approach. Environ. Sci. Policy.

[8] Moreira F., Rego F.C., Ferreira P.G. (2001). Temporal (1958–1995) pattern of change in a cultural landscape of northwestern Portugal: Implications for fire occurrence. Landsc. Ecol..

[9] Schmitz M.F., De Aranzabal I., Aguilera P.A., Rescia A., Pineda F.D. (2003). Relationship between landscape typology and socioeconomic structure: Scenarios of change in Spanish cultural landscapes. Ecol. Model..

[10] Peña J., Bonet A., Bellot J., Sánchez J., Eisenhuth D., Hallett S., Aledo A., Koomen E., Stillwell J., Bakema A., Scholten H.J. (2007). Driving Forces of Land-Use Change in a Cultural Landscape of Spain. Modelling Land-Use Change: Progress and Applications.

[11] De Aranzabal I., Schmitz M.F., Aguilera P., Pineda F.D. (2008). Modelling of landscape changes derived from the dynamics of socio-ecological systems: A case of study in a semiarid Mediterranean landscape. Ecol. Indic..

[12] Álvarez Martínez J., Suárez-Seoane S., De Luis Calbuig E. (2011). Modelling the risk of land cover change from environmental and socio-economic drivers in heterogeneous and changing landscapes: The role of uncertainty. Landsc. Urban Plan..

[13] Schmitz M., Matos D., De Aranzabal I., Ruíz-Labourdette D., Pineda F. (2012). Effects of a protected area on land-use dynamics and socioeconomic development of local populations. Biol. Conserv..

[14] Müller F., Burkhard B. (2012). The indicator side of ecosystem services. Ecosyst. Serv..

[15] Pearl J. (1988). Probabilistic Reasoning in Intelligent Systems.

[16] Shannon C.E. (1948). A mathematical theory of communication. Bell Syst. Tech. J..

[17] Aguilera P.A., Fernández A., Fernández R., Rumí R., Salmerón A. (2011). Bayesian networks in environmental modelling. Environ. Model. Softw..

[18] Landuyt D., Broekx S., D’hondt R., Engelen G., Aertsens J., Goethals P. (2013). A review of Bayesian belief networks in ecosystem service modelling. Environ. Model. Softw..

[19] Korb K.B., Nicholson A.E. (2003). Bayesian Artificial Intelligence.

[20] Maldonado A.D., Aguilera P.A., Salmerón A., Nicholson A.E. (2018). Probabilistic modeling of the relationship between socioeconomy and ecosystem services in cultural landscapes. Ecosyst. Serv..

[21] Friedman N., Geiger D., Goldszmidt M. (1997). Bayesian Network Classifiers. Mach. Learn..

[22] Scutari M. (2010). Learning Bayesian Networks with the bnlearn R Package. J. Stat. Softw..

